# Nanoparticle Adjuvant Design Enhances Germinal Center Responses Targeting Conserved Subdominant Epitopes for Pan‐Coronavirus Vaccine Development

**DOI:** 10.1002/advs.202512100

**Published:** 2025-11-25

**Authors:** Sijin Huang, Kanella M. Cohen, Liqiang Chen, Xiaowo Kang, Chang Liu, Megan E. Demouth, Wenxia Jiang, Alexander R. Maldeney, Rong Tong, Zunlong Ke, Kartik Chandran, Wei Luo, Qian Yin

**Affiliations:** ^1^ Department of Biomedical Engineering The University of Texas at Austin Austin TX 78712 USA; ^2^ Department of Molecular Biosciences The University of Texas at Austin Austin TX 78712 USA; ^3^ Department of Microbiology and Immunology Albert Einstein College of Medicine New York NY 10461 USA; ^4^ Department of Microbiology and Immunology Indiana University School of Medicine Indianapolis IN 46202 USA; ^5^ Department of Chemical Engineering Virginia Polytechnic Institute and State University Blacksburg VA 24061 USA; ^6^ LaMontagne Center for Infectious Diseases The University of Texas at Austin Austin TX 78712 USA; ^7^ Texas Materials Institute University of Texas at Austin Austin TX 78712 USA

**Keywords:** antibody breadth and durability, germinal center, nanoparticle adjuvant, Pan‐coronavirus vaccine, SARS‐CoV‐2

## Abstract

Current SARS‐CoV‐2 vaccines primarily elicit antibodies targeting the variable receptor‐binding domain in the S1 subunit of the spike protein, resulting in limited cross‐reactivity and short‐lived immunity against emerging variants. The conserved S2 subunit presents a promising vaccine target for broad and durable protection, but the immunodominance in vaccine‐induced germinal center (GC) responses hinders effective antibody generation against S2. Here, a polymeric toll‐like receptor 7 agonist nanoparticle (TLR7‐NP) adjuvant is reported, well designed to enhance lymph node targeting and more efficiently activate S2‐specific B cells. When combined with Alum‐adsorbed SARS‐CoV‐2 HexaPro spike protein, TLR7‐NP promotes early GC recruitment of S2‐specific B cells and overcomes the immunodominance, leading to early and robust S2‐specific antibody responses. Compared to conventional TLR7‐Alum adjuvanted subunit vaccine and clinically used SARS‐CoV‐2 mRNA vaccine, TLR7‐NP adjuvant induces stronger humoral immune responses across sarbecoviruses and betacoronaviruses and promotes long‐lived plasma cell and memory B cell formation. These findings present a direct B cell‐activating adjuvant approach for effective pan‐coronavirus vaccine development.

## Graphical abstract

Current SARS‐CoV‐2 vaccines generate short‐lived, strain‐specific immunity. A polymeric TLR7 agonist nanoparticle (TLR7‐NP) adjuvant enhances lymph node targeting and promotes early and sustained germinal center responses toward conserved S2 epitopes of the spike protein. This approach elicits broad and durable humoral immune responses, representing a promising platform for next‐generation pan‐coronavirus vaccine development.

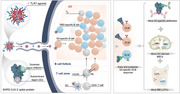

## 1 Introduction

Vaccines remain one of the most effective strategies to prevent infectious diseases and preserve public health through inducing protective antibody responses. The rapid development and deployment of SARS‐CoV‐2 vaccines have successfully mitigated the severity and spread of viruses, significantly reducing hospitalizations and mortality worldwide. However, a major limitation of current SARS‐CoV‐2 vaccines is their narrow protection and short‐lived immunity.^[^ ^1^, ^2^, ^3^, ^4^ ^]^ Antibody responses elicited by these vaccines predominantly target highly variable receptor‐binding domain (RBD) of the spike protein,^[^ ^5^, ^6^ ^]^ resulting in limited cross‐reactivity against emerging variants and other human coronaviruses. Consequently, frequent boost vaccination and updated formulation are required to maintain protection, highlighting the urgent need to develop a pan‐coronavirus vaccine capable of inducing broad and durable antibody responses across diverse coronaviruses.

Coronavirus vaccines can be divided into three levels of breadth based on their protection ranges:^[^ ^7^ ^]^ 1) primary level: targeting all sarbecoviruses (e.g., SARS‐CoV and SARS‐CoV‐2), 2) middle level: betacoronaviruses (e.g., MERS‐CoV and endemic strains like HCoV‐OC43), and 3) high level: targeting all coronaviruses. Results have shown that neutralizing antibodies in currently vaccinated human sera mainly target S1 subunit, particularly the RBD and N‐terminal domain (NTD), which are prone to antigenic drift due to selective immune pressure. In contrast, the S2 subunit is highly conserved across multiple strains and contains functionally critical regions involved in viral membrane fusion. Antibodies targeting S2, including non‐neutralizing antibodies, have demonstrated cross‐reactivity against spike proteins from diverse betacoronaviruses.^[^ ^8^, ^9^, ^10^, ^11^ ^]^ Even non‐neutralizing antibodies targeting S2 can exhibit protective FcR‐mediated effector functions across diverse sarbecoviruses and variants of concern.^[^ ^12^, ^13^ ^]^ Thus, eliciting robust antibody responses against the conserved S2 region offers a promising path toward pan‐coronavirus immunity.

Despite its promise, successfully eliciting potent S2‐specific antibody responses has proven challenging using current vaccination approaches. The S2 subunit, while conserved, is typically immunologically subdominant compared to the highly immunogenic S1 subunit.^[^ ^5^, ^14^ ^]^ Following vaccination, the quality and durability of vaccine‐elicited humoral responses are governed by germinal center (GC) responses,^[^ ^15^, ^16^ ^]^ where antigen‐specific B cells undergo clonal expansion, somatic hypermutation to develop higher‐affinity antibodies. These B cells then exit GCs to differentiate into long‐lived plasma cells (LLPCs) or memory B cells (MBCs), contributing to lasting humoral immunity for long‐term protection. However, during GC competition, B cell clones targeting dominant and variable epitopes in S1 often outcompete those recognizing conserved yet subdominant regions like S2.^[^ ^6^, ^17^ ^]^ This immunodominance limits the development of broad humoral immune responses. Therefore, strategies that can modulate GC responses to promote the recruitment and maturation of S2‐specific B cells are critical for the rational design of pan‐coronavirus vaccines.

To date, efforts to enhance GC responses to subdominant epitopes have largely focused on antigen design. For example, prefusion‐stabilized SARS‐CoV‐2 S2‐only proteins^[^ ^18^ ^]^ or multivalent S2‐based vaccine^[^ ^19^ ^]^ have been developed to increase the visibility of S2 region. While antigen remains important, adjuvants also play a crucial yet underexplored role in shaping the diversity and quality of GC responses.^[^ ^20^ ^]^ Traditional adjuvant functions by activating the innate immune system, primarily dendritic cells (DCs), which then orchestrates follicular T cell (Tfh) responses to support GC B cell selection and effector differentiation through IL‐21 and CD40L signaling.^[^ ^21^ ^]^ Most adjuvants have been designed with DCs as the primary target, but strategies to directly engage B cells to influence GC outcomes remain largely unexplored.

**Scheme 1:**
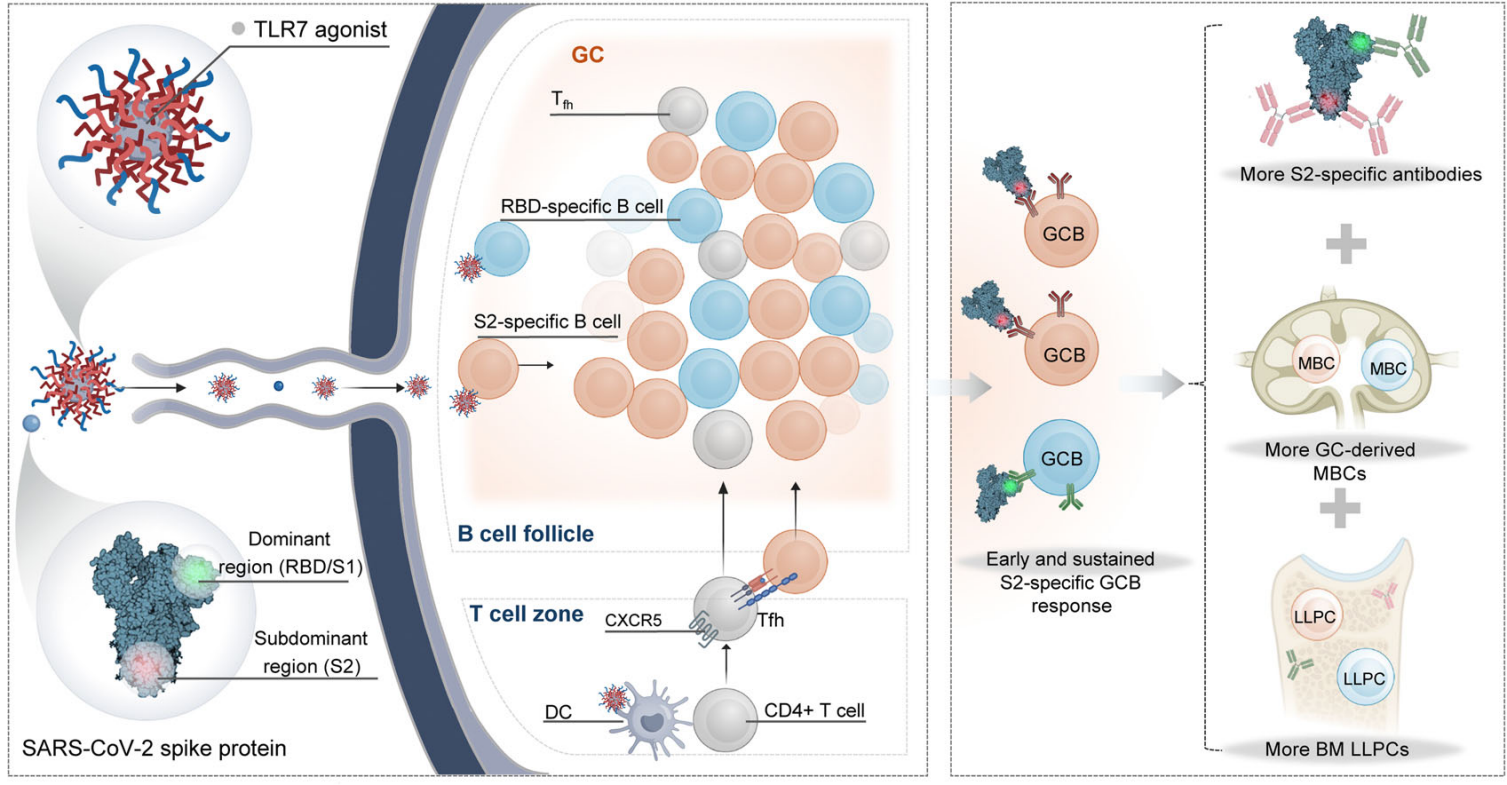
Nanoparticle adjuvant design overcomes the immunodominance and promotes GC responses targeting the conserved S2 subunit of the SARS‐CoV‐2 spike protein. This strategy employs a TLR7 agonist‐loaded nanoparticle (TLR7‐NP) adjuvant to enhance lymph node delivery and directly activate S2‐specific B cells, thereby modulating GC responses and overcome immunodominance to induce more S2‐specific antibodies. This approach boosts cross‐reactive humoral immune responses and promotes more generation of long‐lived plasma cell and memory B cell formation, paving a way for the development of effective pan‐coronavirus vaccines.

To address this gap, we previously developed a TLR7 agonist‐loaded nanoparticle (TLR7‐NP) adjuvant platform with well‐controlled physicochemical properties and controlled agonist release kinetics.^[^ ^22^ ^]^ In the current study, we leverage this established platform in combination with SARS‐CoV‐2 spike protein to uncover new mechanistic insights into how direct B cell‐intrinsic TLR7 activation reshapes the GC and humoral immune responses (**Scheme** **1**). TLR7‐NPs promoted efficient accumulation in the draining lymph node and enhanced direct uptake by B cells, which collectively drives potent TLR7 signaling and early B cell activation. Compared to conventional TLR7 agonist adsorbed on aluminum hydroxide (TLR7‐Alum), TLR7‐NP‐adjuvanted vaccination significantly enhanced S2‐specific B cell activation, promoted their early GC recruitment, and expanded S2‐specific GC B cell and antibody responses with broad cross‐reactivity across sarbecoviruses and betacoronaviruses, outperforming responses elicited by a clinically used SARS‐CoV‐2 mRNA vaccine. Furthermore, this strategy markedly increased the generation of LLPCs and MBCs, resulting in durable antibody responses against multiple coronavirus variants that persisted for at least four months post immunization.

These findings represent a critical advance in adjuvant design, demonstrating that direct B cell‐targeted activation via nanoparticle delivery can reshape GC immunodominance and promote broadly reactive humoral immunity. The approach provides a promising strategy for next‐generation pan‐coronavirus vaccine development.

## 2 Results

### 2.1 TLR7‐NP Exhibits Well‐Controlled Physicochemical Properties and Sustained TLR7 Activation In Vitro

**Figure 1:**
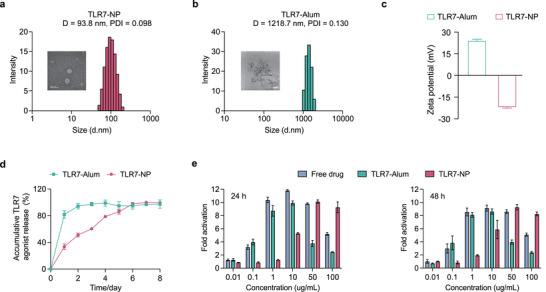
Physicochemical characterization and in vitro TLR7 activation of TLR7‐NP and TLR7‐Alum. a,b) Hydrodynamic size and size distribution of (a) TLR7‐NP and (b) TLR7‐Alum measured by dynamic light scattering (DLS); representative morphology of TLR7‐NP visualized by negative staining transmission electron microscopy (TEM) and TLR7‐Alum by cryo‐electron microscopy (cryo‐EM). c) Zeta potential measurements of TLR7‐NP and TLR7‐Alum. d) In vitro release kinetics of TLR7 agonist from TLR7‐NP and TLR7‐Alum in PBS. e) In vitro TLR7 activation assessed using HEK‐Blue mTLR7 reporter cells treated with equivalent doses of free agonist, TLR7‐Alum or TLR7‐NP over 24 h and 48 h (n = 3). Data are represented as mean ± SD.

TLR7‐NPs were synthesized as previously described,^[^ ^22^ ^]^ via TLR7 agonist (gardiquimod)‐initiated ring‐opening polymerization, followed by nanoprecipitation with poly(ethylene glycol)‐*b*‐poly(lactic‐co‐glycolic acid) (PEG‐PLGA) (Figure S1a, Supporting Information). This method formulates nanoparticles with well‐controlled physicochemical properties, including tunable size and size distribution, surface charge and drug release kinetics. Unlike conventional TLR7‐Alum formulations (Figure S1b, Supporting Information), where the TLR7 agonist is physically adsorbed onto aluminum hydroxide forming ≈1.2 µm aggregates, TLR7‐NP exhibits a significantly smaller particle size (≈94 nm) with a narrow size distribution (PDI = 0.098) (**Figure** **1**a,b). This nanoparticle size facilitates efficient passive drainage into the lymphatic system and accumulation in draining lymph nodes, the primary sites of immune priming. The zeta potential of TLR7‐NP is moderately negative (−22mV), a surface feature that will help reduce nonspecific protein adsorption, enhance colloidal stability and promote lymphatic trafficking upon administration (Figure 1c; Figure S2, Supporting Information). In contrast, the positively charged TLR7‐Alum tends to bind serum proteins and aggregate, potentially limiting their mobility and bioavailability.

In addition to its favorable size and surface properties, TLR7‐NP exhibits sustained release of the TLR7 agonist over seven days, in sharp contrast to the burst release from TLR7‐Alum, which releases ≈80% of the agonist within the first 24 h (Figure 1d; Figure S3, Supporting Information). This sustained release profile is particularly important for maintaining prolonged immune stimulation and better mimicking the natural viral infection. The functional relevance of this release profile was further confirmed using an in vitro TLR7 reporter cell assay (Figure 1e). At 24 h, free TLR7 agonist and TLR7‐Alum showed a biphasic dose‐response peaking at 1–10 µg mL^−1^ but declining sharply at higher concentrations, consistent with rapid receptor occupancy followed by desensitization. In contrast, TLR7‐NP showed limited activation activity below 1 µg mL^−1^ but induced progressively stronger and more sustained activation at 10–100 µg mL^−1^. Notably, at 50–100 µg mL^−1^, TLR7‐NP maintained high activation activity from 24 and 48 h, whereas responses from free agonist and TLR7‐Alum already diminished. This sustained activity likely results from NP‐mediated intracellular delivery of the agonist and its gradual release via ester bond cleavage, which provides prolonged receptor engagement and downstream activation.

### 2.2 TLR7‐NP Enhances Draining Lymph Node Accumulation, Antigen Presenting Cell Uptake, and S2‐Specific B Cell Activation

**Figure 2:**
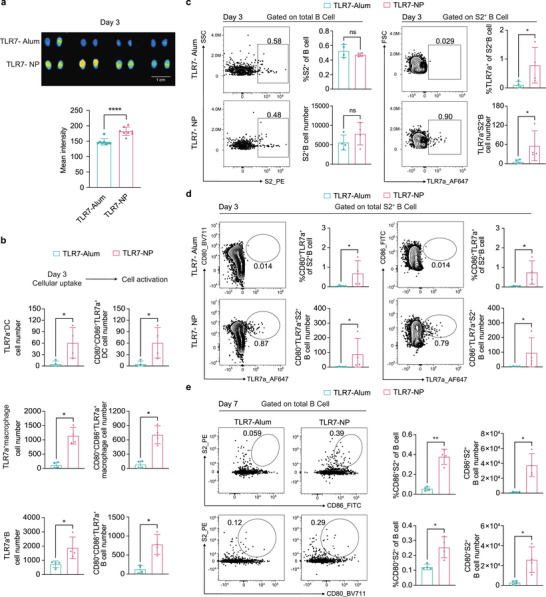
TLR7‐NP enhances draining lymph node accumulation, antigen‐presenting cell internalization, and S2‐specific B cell activation. C57BL/6 mice were immunized with Alum‐adsorbed SARS‐CoV‐2 HexaPro (HexaPro‐Alum,10 µg) combined with either TLR7‐Alum or TLR7‐NP at day 0. In panels (a–d), TLR7 agonist (gardiquimod) was labeled with AF647 (excitation/emission = 651/672 nm) fluorophore, and an equivalent dose of AF647‐TLR7 agonist (20 nmol) was used in both TLR7‐Alum and TLR7‐NP. In panel (e), TLR7 agonist (gardiquimod) was administered at an equivalent dose (20 µg) without AF647 in both TLR7‐Alum and TLR7‐NP. a) Representative fluorescence images and quantification of AF647 signal intensity in draining lymph nodes (dLNs) at day 3 (n = 4 mice per group). b) Flow cytometry quantification of AF647‐TLR7 agonist uptake by antigen‐presenting cells in dLNs at day 3. Shown are cell number of AF647‐TLR7 agonist^+^ dendritic cells (TLR7a^+^DCs), macrophage (TLR7a^+^macrophage), and B cells (TLR7a^+^B) (n = 4 mice per group). c) Representative flow cytometry plots and quantification of the frequency and number of S2‐specific B cells (S2^+^B) and S2‐specific B cells that internalized AF647‐TLR7 agonist (TLR7a^+^S2^+^B) in dLNs at day 3 (n = 4 mice per group). d) Representative flow cytometry plots and quantification of activated S2‐specific B cells that internalized AF647‐TLR7 agonist in dLNs at day 3. Shown are the percentage and cell numbers of CD80^+^TLR7a^+^S2^+^B cells and CD86^+^ TLR7a^+^S2^+^B cells (n = 4 mice per group). e) Representative flow cytometry plots and quantification of activated S2‐specific B cells in dLNs at day 7. Shown are the percentage and cell numbers of CD80^+^S2^+^B cells and CD86^+^S2^+^B cells (n = 4 mice per group). Data are represented as mean±SD and analyzed by Mann‐Whitney test. ^*^ *p* < 0.05, ^**^ *p* < 0.01, ^***^ *p* < 0.001.

To assess how the physicochemical properties of TLR7‐NP impact its biodistribution and interaction with the immune system across tissue and cellular scales, we administered AlexaFluor647 (AF647)‐labeled TLR7 agonist formulated as either TLR7‐NP or TLR7‐Alum, in combination with alum‐adsorbed SARS‐CoV‐2 HexaPro protein, into C57BL/6 mice. Draining lymph nodes were harvested at 3 days post injection to assess the accumulation of the labeled TLR7 agonist via fluorescence intensity of AF647. As shown in **Figure** **2**a, TLR7‐NP induced significantly higher fluorescence intensity in draining lymph nodes than TLR7‐Alum, indicating enhanced lymph node accumulation and retention.

At the cellular level, we further investigated how the TLR7‐NP engaged immune cells within draining lymph nodes. Flow cytometry analysis revealed that TLR7‐NP was more efficiently internalized by all three major antigen‐presenting cells (APCs), including dendritic cells, macrophages, and B cells, than TLR7‐Alum at day 3 (Figure 2b). This enhanced uptake was associated with increased expression of costimulatory molecules CD80 and CD86, indicating potent activation of these APCs (Figure 2b). Using a fluorochrome labeled tetramer probe,^[^ ^23^ ^]^ we next examined B cells targeting the subdominant S2 region, which is typically less immunogenetic during vaccine‐induced humoral immune responses. Although the frequency of S2‐specific B cells was comparable between TLR7‐NP and TLR7‐Alum groups, TLR7‐NP led to markedly higher uptake of TLR7 agonist by S2‐specific B cells (Figure 2c). Critically, this increased uptake was associated with significantly enhanced activation of S2‐specific B cells, as evidenced by upregulation of CD80 and CD86 at day 3 (Figure 2d). This enhanced activation of S2‐specific B cells remained significantly higher in the TLR7‐NP group and persisted though day 7 (Figure 2e). A similar pattern of activation was also observed in RBD‐specific B cells (Figure S4, Supporting Information). These findings suggest that TLR7‐NP significantly improves the cellular uptake of TLR7 agonist by APCs and antigen‐specific B cells, promoting more robust and sustained activation of subdominant S2‐specific B cells.

### 2.3 TLR7‐NP Adjuvanted Immunization Enhances Early and Sustained S2‐Specific GC Responses

**Figure 3:**
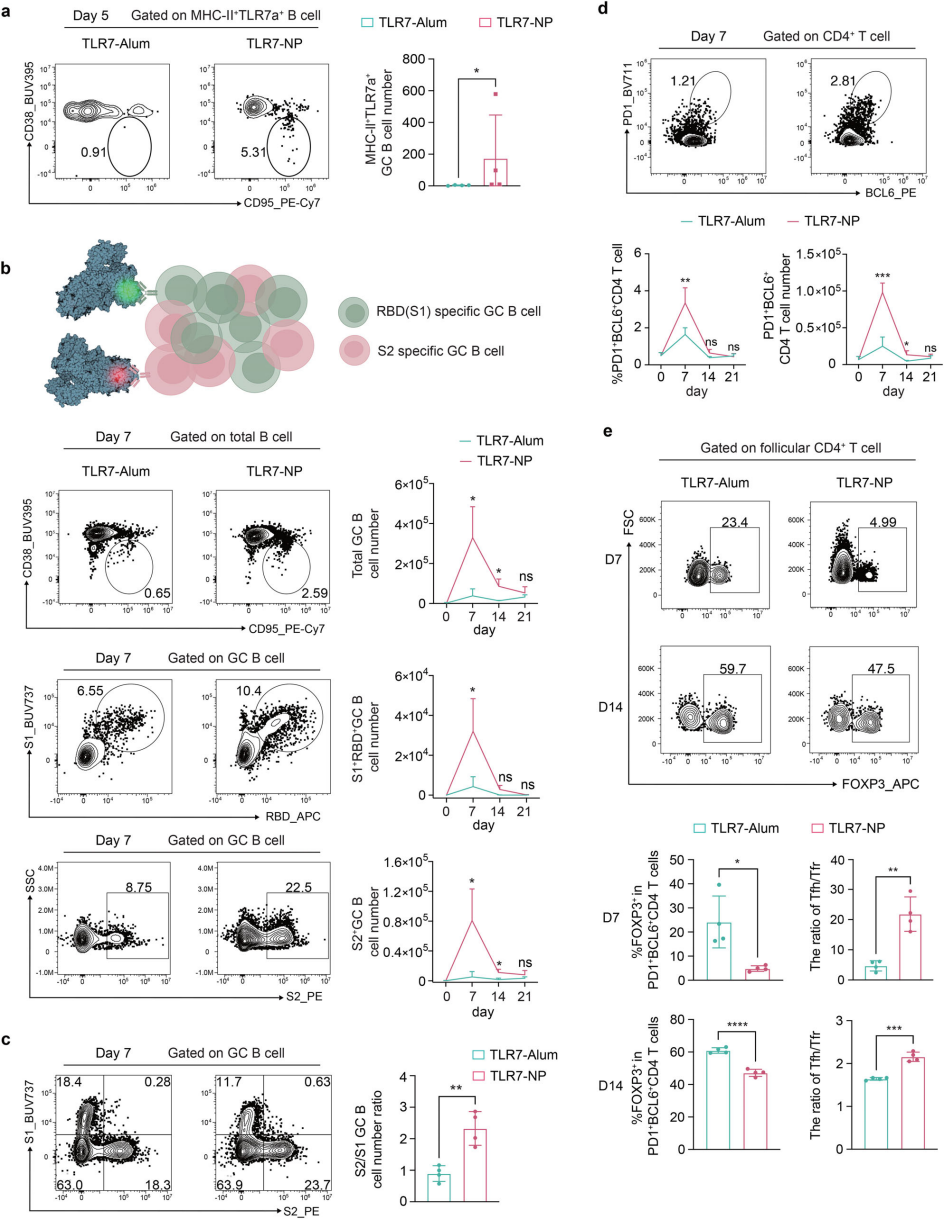
TLR7‐NP enhances the magnitude and quality of GC responses, including those targeting the conserved S2 subunit. C57BL/6 mice were immunized with Alum‐adsorbed SARS‐CoV‐2 HexaPro (HexaPro‐Alum, 10 µg) combined with either TLR7‐Alum or TLR7‐NP at day 0. In panel (a), TLR7 agonist (gardiquimod) was labeled with AF647 (excitation/emission = 651/672 nm) fluorophore, and an equivalent dose of AF647‐TLR7 agonist (20 nmol) was used in both TLR7‐Alum and TLR7‐NP. In panels (b‐e), a TLR7 agonist (gardiquimod) was administered at an equivalent dose (20 µg) in both TLR7‐Alum and TLR7‐NP. a) Representative flow cytometry plots and quantification of early GC B cells derived from activated B cells that internalized AF647‐TLR7 agonist (MHC‐II^+^TLR7a^+^B) in dLNs at day 5 (n=4 mice per group). b) Schematic illustration of antigen‐specific GCB cells and representative flow cytometry plots and kinetics of total GC B cells, RBD(S1)‐specific GC B cells (S1^+^RBD^+^GC B), and S2‐specific GC B cells (S2^+^GC B) in dLNs at days 7, 14, 21 (n=4 mice per group per time point). c) Representative flow cytometry plots and quantification of the ratio of S2‐specific GC B cells (S2^+^GC B) to S1‐specific GC B cells (S1^+^RBD^+^GC B) in dLNs at day 7 (n=4 mice for group). d) Flow cytometry plots and kinetics of Tfh (PD1^+^BCL6^+^CD4^+^) cell number in dLNs at days 7, 14, 21 (n=4 mice for group per time point). e) Representative flow cytometry plots and quantification of Tfr (FOXP3^+^PD1^+^BCL6^+^CD4^+^) cells and Tfh/Tfr ratio in dLNs at days 7 and 14 (n=4 mice for group per time point). All data are represented as mean±SD and analyzed by Mann‐Whitney test. ^*^ *p* < 0.05, ^**^ *p* < 0.01, ^***^ *p* < 0.001.

Vaccine efficacy largely depends on the magnitude and quality of GC responses, which selects for high‐affinity antibody producing B cells and the formation of durable B cell memory.^[^ ^15^, ^24^ ^]^ Given the ability of TLR7‐NP to promote stronger and sustained B cell activation, we investigated how this adjuvant formulation influenced the early recruitment of B cells into GCs and shapes the early phase of GC responses. Using AF647 fluorescently labeled TLR7‐NPs, we observed that B cells directly interacting with nanoparticles and exhibiting high MHC‐II expression (TLR7^+^MHC‐II^+^) were recruited into GCs as early as day 5 post‐immunization, indicating TLR7‐NP promoted accelerated GC seeding by directly activating B cells (**Figure** **3**a). To assess how this early activation influenced GC dynamics and antigen‐specificity (Figure 3b), we performed a time‐course study from day 7 to day 21 following immunization with alum‐adsorbed SARS‐CoV‐2 HexaPro spike protein (HexaPro‐Alum) in combination with either TLR7‐Alum or TLR7‐NP. As shown in Figure 3b, TLR7‐NP immunization significantly boosted early GC responses, with markedly increased total GC B cell number in the draining lymph node at day 7 compared to TLR7‐Alum, and this enhancement was maintained through day 14. Compared to TLR7‐Alum, TLR7‐NP significantly enhanced GC B cell responses against both the immunodominant RBD epitope in the S1 region (RBD^+^S1^+^) and the conserved but subdominant S2 region (S2^+^) at day 7 and day 14 (Figure 3b). Strikingly, we observed a marked shift in the immunodominance hierarchy in the TLR7‐NP group, with a greater proportion of GC B cells targeting the conserved S2 region relative to the typically dominant S1 region (Figure 3c).

GC responses rely on B‐T cell interactions,^[^ ^25^ ^]^ where T follicular helper (Tfh) cells provide essential signals to support GC B cells survival and selection, while T follicular regulatory cells (Tfr) restrain GC reaction. TLR7‐NP immunization led to a marked increase in both the frequency and the absolute number of Tfh cells (Figure 3d), accompanied by a significant reduction in the proportion of Tfr cells from 23.4% to 4.99% compared to TLR7‐Alum (Figure 3e). This shift resulted in a substantially elevated Tfh/Tfr ratio at day 7, which was sustained though day 14 (Figure 3e). Collectively, these results demonstrated that TLR7‐NP not only accelerates GC formation but also promotes sustained GC B cell responses targeting the subdominant S2 region, which is likely further supported by the enhanced Tfh help and reduced Tfr suppression.

To determine whether the enhanced GC responses were attributable to the TLR7 ligand or the nanoparticle carrier, we immunized mice with HexaPro‐Alum adjuvanted with either TLR7‐NP or control nanoparticles lacking the TLR7 ligand (mPEG‐PLA NP). As shown in Figure S5 (Supporting Information), the TLR7‐NP–adjuvanted group exhibited significantly higher total and antigen‐specific (S1^+^RBD^+^ and S2^+^) GC B cell numbers at day 7 compared to the control nanoparticle group. No significant difference was observed between the non‐adjuvanted HexaPro group (black) and the HexaPro‐Alum + mPEG‐PLA NP group, indicating that the adjuvant effect derived from the TLR7 ligand rather than the nanoparticle carrier. The frequency of CXCR5^+^PD1^+^ follicular CD4^+^ T cells followed the same trend, further confirming that TLR7 signaling mediated the enhanced GC and Tfh responses. To dissect the contributions of adjuvant and antigen formulations, we first immunized mice with HexaPro‐Alum alone, HexaPro‐Alum + free TLR7, HexaPro + TLR7‐NP, or HexaPro‐Alum + TLR7‐NP. As shown in Figure S5 (Supporting Information), only nanoparticle formulated TLR7 ligand but not the free TLR7 ligand enhanced GC B cell responses compared to HexaPro‐Alum alone, confirming the critical role of nanoparticle formulation in adjuvant activity. We then compared different antigen formulations (HexaPro vs. HexaPo‐Alum) in combination with TLR7‐NP. Although HexaPro + TLR7‐NP induced measurable GC responses, they were lower than those elicited by HexaPro‐Alum + TLR7‐NP, suggesting that Alum further promoted GC formation, likely by enhancing antigen retention.^[^ ^26^, ^27^ ^]^ A similar trend was observed for Tfh response (Figure S5, Supporting Information). Together, these results demonstrate that TLR7‐NP and Alum act synergistically to elicit robust GC and Tfh responses, with the nanoparticle‐formulated TLR7 ligand playing as key determinant of adjuvant activity.

### 2.4 TLR7‐NP Adjuvanted Immunization Induces Rapid and Potent S2‐Specific Antibody Responses

**Figure 4:**
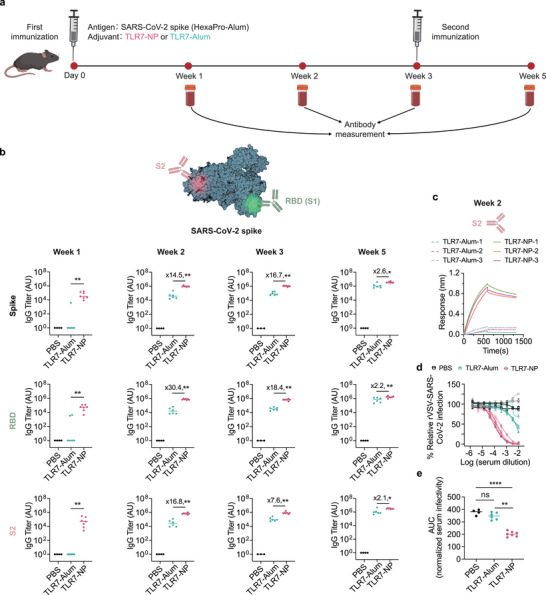
TLR7‐NP enhances antibody responses targeting multiple domains of SARS‐CoV‐2 HexaPro spike protein, including RBD(S1), S2 and full‐length spike. a) Immunization and sample collection schedule. C57BL/6 mice were immunized with Alum‐adsorbed SARS‐CoV‐2 HexaPro spike protein (HexaPro‐Alum) combined with either TLR7‐NP or TLR7‐Alum at weeks 0 and 3. Serum samples were collected at weeks 1, 2, 3, and 5 post primary immunization. b) Antigen‐specific IgG titers were quantified by ELISA at indicated time points. Data are shown as medians with each dot representing one mouse (*n* = 6, 7 mice for TLR7‐Alum, TLR7‐NP). Data are analyzed by Mann–Whitney test. ^*^ *p* < 0.05, ^**^ *p* < 0.01, ^***^ *p* < 0.001. c) Binding kinetics of serum antibodies to the S2 measured by bio‐layer interferometry (BLI). BLI binding curves of serum samples collected at week 2 diluted 1:100. d) Representative neutralization curves using week 2 sera, plotting % relative rVSV‐SARS‐CoV‐2 infection versus log10 serum dilution for PBS, TLR7‐Alum, and TLR7‐NP groups. e) Neutralization activity of sera collected at week 2 post‐immunization, shown as normalized area under the curve (AUC) of serum infectivity inhibition against rVSV‐SARS‐CoV‐2 infection. Data are shown as medians with each dot representing one mouse (n = 6, 7 mice for TLR7‐Alum, TLR7‐NP). Data are analyzed by Welch's *t*‐test. ^*^ *p* < 0.05, ^**^ *p* < 0.01, ^***^ *p* < 0.001, ^****^ *p* < 0.0001, ns= not significant.

Early GC seeding enhances the recruitment of S2‐specific B cell clones, while the sustained increase in the ratio of Tfh to Tfr from day 7 to day 14 could enhance help signals that support the survival and maturation of these subdominant clones. Together, these TLR7‐NP‐induced effects suggest their potential to overcome immunodominance and promote broad antibody responses targeting conserved S2 epitopes during vaccine‐induced immunity. To explore this possibility, we immunized mice with alum‐adsorbed SARS‐CoV‐2 HexaPro spike protein in combination with either TLR7‐NPs or TLR7‐Alum at week 0 and 3, followed by a time‐course serological analysis at weeks 1, 2, 3, and 5 post primary immunization (**Figure** **4**a). TLR7‐NPs significantly increased antibody titers against whole spike as well as various epitopes including RBD and S2 as early as week 1, and these enhanced responses were maintained through week 5 (Figure 4b). Notably, 100% of mice immunized with TLR7‐NP adjuvanted HexaPro‐Alum developed detectable IgG antibodies against the conserved S2 region, a region typically difficult to target. In contrast, none of mice receiving TLR7‐Alum adjuvanted HexaPro‐Alum generated detectable S2‐specific IgG antibodies at week 1. Given that affinity maturation is a critical feature of GC responses, we next assessed the binding quality of S2‐specific IgG antibodies generated in the different immunization groups using bio‐layer interferometry (BLI). Despite the presence of S2‐specific antibodies in both TLR7‐NP and TLR7‐Alum groups by week 2, the TLR7‐NP group showed consistently higher maximum binding responses at comparable serum dilutions (Figure 4c). These results suggest that TLR7‐NP immunization induced a higher concentration of S2‐specific antibodies compared to TLR7‐Alum. To further evaluate the functional quality of antibody responses, we performed an rVSV‐SARS‐CoV‐2 neutralization assay using serum samples collected at week 2 (two weeks post‐prime) and week 5 (two weeks post‐boost) from mice immunized with HexaPro‐Alum adjuvanted with either TLR7‐Alum or TLR7‐NP. As shown in Figure 4d,e, serum samples from the TLR7‐NP group exhibited markedly enhanced neutralizing activity as early as week 2, with ≈2‐log higher neutralization titers and significantly reduced area under the curve (AUC) values compared to the TLR7‐Alum group. Notably, the TLR7‐NP‐immunized mice also showed significant neutralization activity relative to unvaccinated controls (PBS), whereas the TLR7‐Alum group did not differ from the baseline. By week 5, neutralization titers between the two groups became comparable (Figure S6, Supporting Information). These findings indicate that TLR7‐NP promotes a faster and more potent induction of functional neutralizing antibodies against SARS‐CoV‐2, suggesting its potential to confer early protection and reduce viral transmission following vaccination.

### 2.5 TLR7‐NP Adjuvanted Immunization Induces Cross‐Reactive Antibody Responses Against Diverse Coronavirus Variants

**Figure 5:**
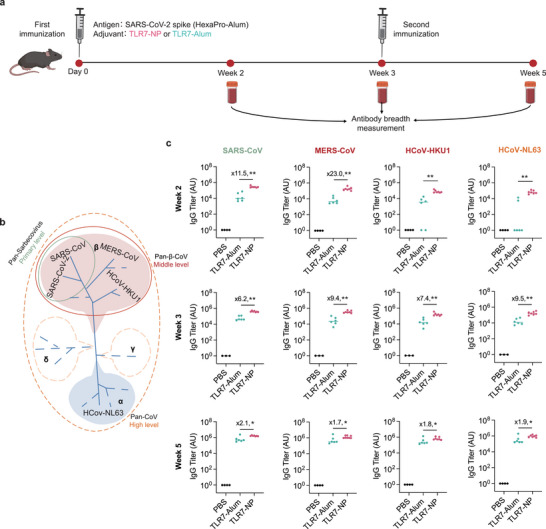
TLR7‐NP‐adjuvanted SARS‐CoV‐2 HexaPro spike vaccine induces cross‐reactive antibody responses against diverse coronavirus variants. a) Immunization and sample collection schedule. C57BL/6 mice were immunized with Alum‐adsorbed SARS‐CoV‐2 HexaPro spike protein combined with either TLR7‐NP or TLR7‐Alum on weeks 0 and 3. Serum samples were collected at Weeks 2, 3, and 5 post primary immunization. b) Phylogenetic tree illustrating different coronaviruses variants at different breadth levels. c) Antigen‐specific IgG titers against spike proteins from various coronavirus variants were quantified by ELISA at indicated time points. Data are shown medians with each dot representing one mouse (*n* = 6, 7 mice for TLR7‐Alum, TLR7‐NP). Data are analyzed by Mann–Whitney test. ^*^ *p* < 0.05, ^**^ *p* < 0.01, ^***^ *p* < 0.001.

The RBD in the S1 region of the coronavirus spike protein is highly variable and mutable, whereas the S2 region is more conserved.^[^ ^5^, ^10^, ^14^ ^]^ We hypothesized that eliciting antibodies targeting the S2 region could enhance the breadth of antibody responses across diverse coronaviruses. To test this possibility, we immunized mice with HexaPro‐Alum, adjuvanted with either TLR7‐NPs or TLR7‐Alum and then assessed antibody responses against spike proteins from heterologous strains from both beta‐ and alpha‐coronavirus groups (**Figure** **5**a,b). The results showed that TLR7‐NP immunization not only increased antibody titers against the SARS‐CoV‐2 spike in the immunized regimen but also induced cross‐reactive antibodies against spike proteins from SARS‐CoV (primary level), MERS‐CoV and HCoV‐HKU1 (middle level), and HCoV‐NL 63 (high level) (Figure 5c). Strikingly, 100% of mice immunized with TLR7‐NP‐adjuvanted HexaPro‐Alum developed high titers of IgG antibodies against HCoV‐NL63 as early as week 2. HCoV‐NL63 belongs to a different group from SARS‐CoV‐2 in the phylogenetic tree and represents as a seasonal human coronavirus that primarily found in young children, the elderly and immunocompromised patients with acute respiratory illness. In contrast, only 33.3% of mice immunized with TLR7‐Alum‐adjuvanted HexaPro‐Alum generated detectable but lower‐titer antibodies at week 2. This striking difference demonstrates the ability of TLR7‐NP immunization to elicit early and robust cross‐reactive antibody responses, highlighting its potential to generate broad‐spectrum protection against diverse coronaviruses and to reduce the burden of respiratory illnesses, particularly in vulnerable populations.

### 2.6 TLR7‐NP–Adjuvanted Vaccine Induces Broader and Stronger Cross‐Reactive Antibody Responses than SARS‐CoV‐2 mRNA Vaccine

**Figure 6:**
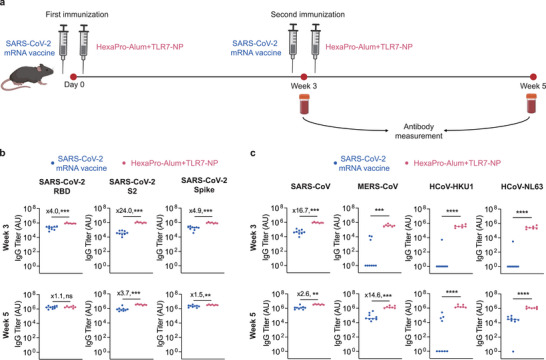
TLR7‐NP‐adjuvanted SARS‐CoV‐2 HexaPro spike vaccine elicits broader and stronger cross‐reactive antibody responses than the clinically used SARS‐CoV‐2 mRNA vaccine. a) Immunization and sample collection schedule. C57BL/6 mice were immunized with Alum‐adsorbed SARS‐CoV‐2 HexaPro spike protein combined with either TLR7‐NP or SARS‐CoV‐2 mRNA vaccine on weeks 0 and 3. Serum samples were collected at weeks 2, 3, and 5 post primary immunization. b,c) Antigen‐specific IgG titers against spike proteins from various coronavirus variants were quantified by ELISA at indicated time points. Data are shown medians with each dot representing one mouse (*n* = 9, 7 mice for SARS‐CoV‐2 mRNA vaccine, TLR7‐NP). Data are analyzed by Mann–Whitney test. ^*^ *p* < 0.05, ^**^ *p* < 0.01, ^***^ *p* < 0.001, ns = not significant.

Limited antibody breadth has been reported with currently licensed mRNA vaccines. To benchmark antibody responses induced by TLR7‐NP‐adjuvanted immunization to those elicited by mRNA vaccination, we immunized mice with either a clinically used SARS‐CoV‐2 mRNA vaccine or TLR7‐NP‐adjuvanted HexaPro‐Alum at weeks 0 and 3 (**Figure** **6**a). We then analyzed antibodies in the sera against the SARS‐CoV‐2 RBD, S2, and full‐length spike as well as Spike proteins from various heterologous coronavirus subtypes (Figure 6b,c). As shown in Figure 6b, all mice immunized with TLR7‐NP‐adjuvanted HexaPro‐Alum developed significantly higher antibody titers against RBD, S2, and Spike of SARS‐CoV‐2 compared to those immunized with the SARS‐CoV‐2 mRNA vaccine, as early as 3 weeks after primary immunization. Notably, S2‐specific and Spike‐specific antibodies remained significantly higher in the TLR7‐NP group through week 5, two weeks post the second immunization. In addition, all mice receiving TLR7‐NP‐adjuvanted HexaPro‐Alum successfully developed robust antibodies against spike proteins from SARS‐CoV‐2, SARS‐CoV, MERS‐CoV, HCoV‐HKU1, and HCoV‐NL63. In sharp contrast, most mice immunized with SARS‐CoV‐2 mRNA vaccine failed to generate detectable cross‐reactive antibodies against HCoV‐HKU1 and HCoV‐NL63, 3 weeks after the first immunization. Even after two immunizations, SARS‐CoV‐2 mRNA vaccinated mice were unable to develop high titers of cross‐reactive antibodies against HCoV‐HKU1 and HCoV‐NL63 as compared to TLR7‐NP‐adjuvanted HexaPro‐Alum group (Figure 6c). These findings further demonstrated TLR7‐NP‐adjuvanted vaccination can significantly broaden the antibody responses against diverse human coronaviruses, highlighting its potential as a novel strategy for pan‐coronavirus vaccine development.

### 2.7 TLR7‐NP Adjuvanted Vaccine Enhances Generation of Long‐Lived Plasma Cell and Memory B Cell

**Figure 7:**
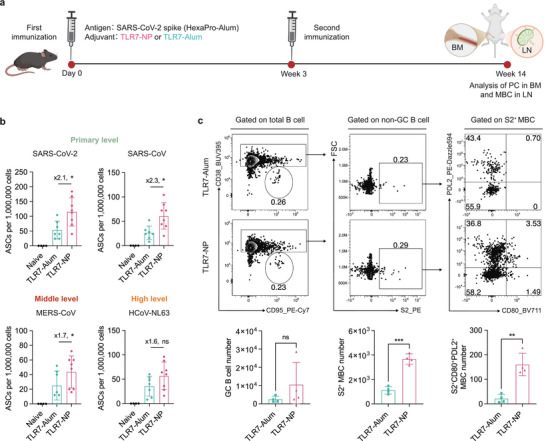
TLR7‐NP promotes durable humoral immunity characterized by enhanced plasma cell and memory B cell responses 14 weeks post‐immunization. a) Immunization and sample collection schedule. C57BL/6 mice were immunized with Alum‐adsorbed SARS‐CoV‐2 HexaPro spike protein combined with either TLR7‐NP or TLR7‐Alum on weeks 0 and 3. Bone marrow and dLNs were collected at week 14. b) Quantification of antibody‐secreting cells (ASCs) specific for SARS‐CoV‐2, SARS‐CoV, MERS‐CoV, and HCoV‐NL63 in the bone marrow by ELISPOT assay. c) Representative flow plots and quantification of S2‐specific memory B cells (S2^+^MBCs) and its CD80^+^PDL2^+^ subsets (S2^+^CD80^+^PDL2^+^MBCs) in dLNs. Data are analyzed by Mann–Whitney test. ^*^ *p* < 0.05, ^**^ *p* < 0.01, ^***^ *p* < 0.001, ns = not significant.

The effector differentiation of GC B cells to LLPCs and MBCs provides long‐term vaccine protection. LLPCs, primarily residing in the bone marrow, sustain a reservoir of high‐affinity circulating antibodies. MBCs, on the other hand, enable rapid humoral recall responses upon antigen re‐exposure. To assess whether TLR7‐NP‐adjuvanted vaccination influences memory formation, we performed ELISPOT assay on bone marrow cells and analyzed MBCs in the draining lymph node 14 weeks after receiving the primary vaccination (**Figure** **7**a).

Results showed the significantly higher numbers of SARS‐CoV‐2 spike‐specific IgG antibody‐secreting cells (ASCs) in the bone marrow of mice immunized with TLR7‐NP compared to those immunized with TLR7‐Alum (Figure 7b). Notably, TLR7‐NP also induced significantly higher numbers of IgG ASCs targeting spike proteins from other coronaviruses, including SARS‐CoV, MERS‐CoV, and HCoV‐NL63, compared to TLR7‐Alum (Figure 7b; Figure S7, Supporting Information). To assess MBCs, we focused on the subdominant S2‐specific population and used CD80 and PD‐L2 as markers to identify MBC subsets capable of rapidly differentiating into antibody‐secreting cells (ASCs).^[^ ^4^ ^]^ GC responses had contracted by this time and showed no significant differences between two groups. Mice receiving TLR7‐NP had a markedly higher number of S2^+^MBCs and S2^+^CD80^+^PDL2^+^ MBCs compared to the TLR7‐Alum group (Figure 7c). Similarly, significant increase in the number of RBD^+^MBCs and RBD^+^CD80^+^PDL2^+^ MBCs were observed in TLR7‐NP group (Figure S8, Supporting Information). Altogether, these results demonstrate that TLR7‐NP significantly promotes the formation of both LLPCs and MBCs, leading to a broad and durable immune memory. These results highlight the potential of TLR7‐NP to support long‐term protection against current and emerging coronavirus threats.

## 3 Discussion

Over the past two decades, three highly pathogenic coronaviruses, SARS‐CoV (2003), MERS‐CoV (2012), and SARS‐CoV‐2 (2019) have emerged, causing significant mortality and global socioeconomic disruption. The COVID‐19 pandemic caused by SARS‐CoV‐2 virus, in particular, has highlighted the urgent need for next‐generation vaccine strategies that provide broad and durable protection against both existing and emerging coronaviruses. Current licensed vaccines, including mRNA and protein subunit platforms, primarily target the S1 region of the spike protein of SARS‐CoV‐2 and elicit antibodies focused on the RBD epitope. However, the RBD is highly variable and prone to accumulating mutations, resulting in markedly reduced vaccine efficacy against new variants. Variant‐specific boosters have provided only transient protection and often fail to elicit robust immune responses that can effectively cover new or divergent strains. This challenge is further exacerbated by immune imprinting, where the immune system preferentially recalls memory responses against earlier viral variants, thereby limiting its ability to respond to novel epitopes.^[^ ^28^, ^29^ ^]^

To address these challenges, there is growing interest in developing pan‐coronavirus vaccines that target conserved regions of the virus, particularly the S2 subunit of the spike protein. However, immune responses to the S2 region are typically subdominant, and strategies to enhance their immunogenicity remain a central challenge. Most current efforts for enhancing the S2‐specific immune responses have focused on antigen design strategies,^[^ ^18^, ^19^, ^30^ ^]^ such as masking or removing the immunodominant RBD/S1 domains to expose the conserved S2 region or using multivalent spike constructs to enhance cross‐reactivity.

In this study, we presented a different approach by engineering adjuvants with optimized physicochemical properties to modulate the spatiotemporal dynamics of vaccine‐induced immunity and enhance GC responses, particularly those targeting conserved S2 region. Specifically, we designed sub‐100 nm nanoparticles with a negatively charged surface to minimize protein adsorption and promote efficient trafficking to the draining lymph nodes. These nanoparticles also exhibited broad uptake by APCs, including S2‐specific B cells. We also covalently linked a TLR7 agonist to the nanoparticles, enabling controlled release and prolonged TLR7 signaling, which is critical for potent and sustained activation of both B cell and DC. These adjuvant design features profoundly enhanced both the magnitude and quality of GC responses, leading to several key immunological benefits: 1) early and sustained activation of subdominant S2‐specific B cells, driven by enhanced TLR7‐NP uptake and sustained release kinetics, promote their early recruitment into GCs; 2) prolonged GC reaction, supported by sustained TLR7 signaling, provided extended window for the affinity maturation of subdominant S2‐specific B cell clones; 3) enhanced B cell and DC activation synergized to promote Tfh differentiation and persistence, which are critical for sustaining GC reaction and supporting the survival of subdominant S2‐specific B cells. When combined with HexaPro‐Alum, a thermally stable full‐length SARS‐CoV‐2 spike protein adsorbed on the Alum, the TLR7‐NP adjuvant elicited early and potent S2‐specific antibody responses with broad cross‐reactivity against spike proteins from diverse coronaviruses. Importantly, this breadth was accompanied by a durable memory compartment, characterized by increased numbers of LLPCs in the bone marrow secreting antibodies with diverse antigen specificities and increased populations of S2‐specific MBCs expressing CD80 and PDL2, which can quickly differentiate into plasmablasts upon antigen re‐encounter.^[^ ^31^ ^]^

Together, these findings illustrate a promising strategy for pan‐coronavirus vaccine development by demonstrating how rational adjuvant design can reshape GC responses to focus antibody maturation toward subdominant yet conserved epitopes. Beyond coronaviruses, this design principle may be broadly applicable to other pathogens where targeting conserved subdominant epitopes is critical for achieving broader and more durable humoral immunity. Our results align with recent work by Rodrigues et al.,^[^ ^26^ ^]^ which showed that nanoparticle‐based adjuvants incorporating a TLR4 agonist (MPLA) enhanced GC formation and promoted long‐lived bone marrow plasma cells and memory B cells following HIV Env immunization. While both studies highlight the capacity of nanoparticle‐based adjuvants to improve the durability of humoral immunity, our work uniquely demonstrates that TLR7‐NP also accelerates the early induction of GC and antibody responses against subdominant viral epitopes, as early as two weeks post‐immunization. Collectively, these findings suggest that nanoparticle adjuvants can enhance not only the breadth and longevity but also the rapid onset of protective humoral responses across diverse vaccine platforms.

This study demonstrates that TLR7‐NP as a potent vaccine adjuvant capable of eliciting early and high‐titer neutralizing antibody (nAb) responses. A large number of independent studies across vaccine platforms, variants, and species, including non‐human primates and humans, has conclusively established that nAb titers are robust and quantitative correlates of protection for SARS‐CoV‐2 and related coronaviruses.^[^ ^32^, ^33^, ^34^, ^35^ ^]^ Accordingly, the strong nAb responses observed here provide clear evidence supporting the protective potential of this vaccine platform. While this study primarily focuses on humoral responses, increased evidence suggests that other aims of the immune system, particularly T cells, play a crucial role in cross‐protection. Several recent studies targeting conserved T cell epitopes have shown promise for developing next‐generation of pan‐coronavirus vaccines.^[^ ^36^, ^37^ ^]^ Given the efficient uptake of TLR7‐NP by DCs and its ability to sustain their activation, it is likely our platform also contributes to antigen‐specific T cell responses. However, the absence of in vivo challenge experiments in the current study limits our ability to fully dissect the interplay between TLR7‐NP‐induced humoral and cellular immunity in mediating vaccine‐elicited protection. Future mechanistic and in vivo challenge studies will be essential to validate and extend these findings, ultimately establishing a more comprehensive understanding of how this TLR7‐NP adjuvant platform can drive broadly protective vaccine responses.

## 4 Experimental Section

### Preparation of TLR7 Agonist Loaded Nanoparticles (TLR7‐NP)

The TLR7‐NP were prepared as previously reported,^[^ ^22^ ^]^ via two main steps:

TLR7‐polylactide (TLR7‐PLA) polymer preparation: TLR7‐PLA was synthesized via a ring‐opening polymerization method. In a glovebox, gardiquimod (8.5 mg, 27.1 µmol; Cat. No. ALX‐420‐040‐M100, Enzo Life Sciences, NY, USA), a potent TLR7 receptor agonist as initiator, was dissolved in anhydrous tetrahydrofuran (THF) and mixed with (BDI‐EI)ZnN(TMS)_2_ (18.5 mg, 29.8 µmol) as catalyst. After stirring the mixture for 30 min, a THF solution of lactide (97.7 mg, 678 µmol) was added dropwise. The reaction proceeded overnight inside the glovebox. Upon complete consumption of lactide, the reaction was quenched by adding a drop of water. The resulting polymer was precipitated with a 1:1 (v/v) hexane/ether mixture (50 mL), collected by centrifugation, and dried under vacuum. The polymer was characterized using MALDI‐TOF mass spectrometry.

Nanoparticle preparation: A DMF solution containing TLR7‐PLA (100 µL, 10 mg mL^−1^) and PEG‐PLGA (100 µL, 20 mg mL^−1^; Cat. No. AK010, Akina Inc, West Lafayette, IN, USA) was added dropwise into vigorously stirred deionized (DI) water (6 mL). After 5 min of stirring, the resulting TLR7‐NPs were washed with DI water and collected via ultrafiltration (20 min, 4100 × g, Ultracel membrane, 10 kDa MWCO, Millipore).

### Characterization of TLR7‐NP, Alum‐Adsorbed TLR7 (TLR7‐Alum), and Adjuvant‐Antigen Mixtures

For size measurement, the hydrodynamic size and size distribution of TLR7‐NP and TLR7‐Alum were measured by dynamic light scattering (DLS). The morphology of TLR7‐NP was visualized by transmission electron microscopy (TEM) and TLR7‐Alum by cryo‐electron microscopy (Cryo‐EM). For surface charge measurements of TLR7‐NP, TLR7‐Alum, TLR7‐NP+HexaPro‐Alum, and TLR7‐Alum+HexaPro‐Alum, zeta potential was measured using a ZETASIZER Advance dynamic light scattering (DLS) system (Malvern Instruments).

### Negative Staining TEM Sample Preparation and Imaging

TLR7‐NP were diluted to a final concentration of 0.2 mg mL^−1^ in ddH_2_O. A 3 µL aliquot of the sample was applied to a copper grid with carbon film support (Electron Microscopy Sciences) and incubated for 30 s. The grid was then washed twice with ddH_2_O, followed by a single wash with 2% uranyl acetate (Electron Microscopy Sciences). It was subsequently incubated in 2% uranyl acetate for 30 s before air drying. Micrographs were collected on a JEOL NEOARM transmission electron microscope operated at 200 kV.

### Cryo‐EM Sample Preparation and Imaging

TLR7‐Alum was diluted to a final concentration of 0.5 mg mL^−1^ in ddH_2_O. Sample vitrification was performed using a GP2 automatic plunge freezer (Leica Microsystems) at 24 °C and 90% humidity. A 3.5 µL aliquot of the sample was applied to treated holey carbon grids (C‐flat, 2/2, 300 mesh Cu), which had been glow‐discharged for 30 s. The grids were blotted for 3 s using standard filter paper and plunge‐frozen in liquid ethane. After vitrification, the grids were stored in liquid nitrogen until imaging. Imaging was performed using a Glacios cryo‐TEM (Thermo Fisher Scientific) operated at 200 kV, and images were acquired on a Falcon IV direct detector camera using counting mode.

### Release Kinetics Measurement

TLR7‐NP and TLR7‐Alum formulations were suspended in 0.5 mL of PBS buffer and incubated at 37 °C, respectively. Multiple parallel samples were prepared for different time points. At each designated time point, samples were centrifuged, and the supernatant was collected. The concentration of released gardiquimod was quantified using a UV‐Vis spectrophotometer (Cytation 3, Agilent) at a wavelength of 321 nm.

### AF647‐Labeled TLR7‐Alum and TLR7‐NP Preparation

The preparation was conducted according to our previously reported method.^[^ ^22^ ^]^ Briefly, the AF647‐NHS ester (0.5 mg, 0.4 µmol; Cat. No. A20006, Thermo Fisher Scientific, Waltham, MA, USA) was dissolved in 10 µL of DI water and mixed with gardiquimod (132 µg, 0.42 µmol) in 2 µL of DMSO. The reaction mixture was stirred overnight at room temperature. The product was purified via high‐performance liquid chromatography (HPLC, LC‐40D, SHIMADZU) and lyophilized to obtain AF647‐labeled gardiquimod. To prepare AF647‐labeled TLR7‐Alum, the AF647‐labeled gardiquimod was mixed with Alhydrogel® (Cat. No. vac‐alu‐50, InvivoGen, San Diego, CA, USA) under gentle stirring. AF647‐labeled TLR7‐NP was prepared as previously reported.^[^ ^22^ ^]^ After overnight stirring, the reaction mixture was combined with PEG‐PLGA, and the resulting nanoparticles were washed with DI water and collected by ultrafiltration.

### In Vitro TLR7 Signaling Assay

HEK‐Blue‐Lucia^TM^ mTLR7 cells (Cat. No. hkd‐mtlr7ni, InvivoGen, InvivoGen, San Diego, CA, USA) were seeded into 96‐well plates at a density of 5000 cells/well in 180 µL of culture medium in triplicate. Each well was treated with 20 µL of either free drug, TLR7‐Alum, or TLR7‐NP formulations at final TLR7 concentrations of 0.01, 0.1, 1, 10, 50, and 100 µg mL^−1^. After 24 and 48 h of incubation, 10 µL of culture supernatant from each well was collected and analyzed for secreted embryonic alkaline phosphatase (SEAP) using the QUANTI‐Blue assay (Cat. No. rep‐qbs, InvivoGen, San Diego, CA, USA). Specifically, 90 µL QUANTI‐Blue reagent was added to each sample and incubated at 37 °C. SEAP levels were determined by measuring optical density at 625 nm using a microplate reader. Activation efficiency was evaluated based on the increase in optical density relative to the negative control treated with PBS.

### Animals

8‐12 weeks old female C57BL/6 mice were purchased from the Jackson Laboratory. Animals were housed in the University of Texas at Austin Animal Facility under federal, state, and NIH guidelines. The study protocol was reviewed and approved by the University Administrative Panel on Laboratory Animal Care (IACUC, AUP‐2024‐00034). Mice used for SARS‐CoV‐2 mRNA vaccine study were 6‐10 weeks old C57BL/6 mice, obtained from the In Vivo Therapeutics Core (IVT) of the Indiana University Simon Cancer Center. Mice were housed under specific‐pathogen‐free conditions and supervised by the Indiana University Institutional Animal Care and Use Committee. All animals used for in each experiment were randomly assigned into different groups, and studies were unblinded.

### Lymph Node Fluorescence Imaging

Female C57BL/6 mice (8–12 weeks; Jackson Laboratory) were immunized subcutaneously (s.c.) at the base of the tail with Alum‐adsorbed SARS‐CoV‐2 HexaPro spike protein formulated with either AF647‐labeled TLR7‐Alum or AF647‐labeled TLR7‐NP (equivalent gardiquimod dose, 20 nmol) in 100 µL of PBS. On day 3 post‐immunization, the inguinal draining lymph nodes (dLNs) were excised, and AF647 fluorescence signals were measured using a FOBI imaging system (Fluor‐i In Vivo, NEO Science). Image analysis was performed using ImageJ software.

### The Preparation of SARS‐CoV‐2 RBD, S1, and S2 Tetramer

Recombinant biotinylated SARS‐CoV‐2 spike protein subunits, RBD (Cat. No. SPD‐C82E9), S1 (Cat. No. S1N‐C82E8), and S2 (Cat. No. S2N‐C52E8, Acro Biosystems, Newark, DE, USA), were each incubated as monomers with fluorophore‐conjugated streptavidin at a 4:1 molar ratio. Specifically, streptavidin‐AF647 (Cat. No. S32357, Invitrogen, Waltham, MA, USA), streptavidin‐BUV737(Cat. No. 564293, BD biosciences, Franklin Lakes, NJ, USA), and streptavidin‐PE (Cat. No. 405203, BioLegend, San Diego, CA, USA) were used for individual labeling reactions. One‐fifth amount of the total amount of fluorophore‐conjugated streptavidin was added to the monomer solution every 10 min at room temperature. Following the stepwise addition, the reaction solutions were kept in 4 °C overnight.

### Flow Cytometry Analysis of Immune Cells

For cellular uptake and activation study, the mice were s.c. immunized at the base of the tail with alum‐absorbed HexaPro mixed with either AF647‐labeled TLR7‐Alum or AF647‐labeled TLR7‐NP (equivalent gardiquimod dose, 20 nmol) in 100 µL PBS. The inguinal dLNs were excised on day 3 or day 7 post‐immunization and dissociated into single‐cell suspensions using the plunger of a 3 mL syringe on a 70 µm cell strainer. Following cell counting, cells were pelleted by centrifugation and stained on ice for 30 min with tetramers of RBD, S1, and S2. After washing with FACS buffer and centrifugation, the other surface markers were stained on ice for 30 min, including Fc‐blocker, LIVE/DEAD Fixable Aqua Dead Cell Stain, CD19, CD3, CD80, CD86, PDCA1, Ly6C, CD103, CD11c, MHC‐II, and CD11b. Flow cytometric data were acquired using a NovoCyte Penteon flow cytometer (Agilent) with NovoExpress software and analyzed using FlowJo v10. The gating strategy for TLR7a^+^ dendritic cells is on single live CD19^−^CD3^−^CD11c^+^MHC‐II^+^TLR7a^+^ cells. TLR7a^+^B cells were gated on single live CD19^+^CD3^−^TLR7a^+^ cells. TLR7a^+^ macrophage were gated on single live CD19^−^CD3^−^MHC‐II^−^CD11b^+^PDCA1^−^Ly6C^−^TLR7a^+^ cells. The gating strategy for activated TLR7a^+^S2^+^ B cells is on single live CD19^+^CD3^−^S2^+^TLR7a^+^CD86^+^ or CD80^+^ cells. For the day 7 group, mice were immunized as described above, and antigen‐specific activated B cells were identified as single, live CD19⁺CD3^−^CD86⁺ or CD80^+^ and S2⁺ or RBD⁺ cells.

For germinal center (GC) study, dLNs were collected on days 5, 7, 14, and 21 post‐immunizations. Mice in the day 5 group received 20 nmol of gardiquimod, while other groups received 20 µg. For B cell analysis, the cells were stained with RBD, S1, and S2 tetramer on ice for 30 min. After washing and centrifugation, the other surface markers were stained on ice for 30 min, including Fc‐blocker, LIVE/DEAD Fixable Aqua Dead Cell Stain, CD19, CD3, CD80, CD86, MHC‐II, CD95, CD38. For T cell analysis, Cells were stimulated in complete T cell medium with eBioscience™ Cell Stimulation Cocktail (plus protein transport inhibitors) (Cat. No. 00‐4975‐03, Invitrogen, Waltham, MA, USA) for 5 h. After stimulation, the cells were collected, washed, blocked with Fc‐blocker and then stained with LIVE/DEAD Fixable Aqua Dead Cell Stain and surface markers, including CD3, TCRβ chain, CD4, and CD279 (PD‐1), and then fixed using eBioscience Foxp3/Transcription Factor Staining Buffer Set (Cat. No. 00‐5523‐00, Thermo Fisher Scientific, Waltham, MA, USA) according to the manufacturer's instructions. Cells were then washed, permeabilized, stained for function markers, including FOXP3 and BCL6. All the data were acquired using a NovoCyte Penteon flow cytometer (Agilent) and analyzed with FlowJo v10. For the gating strategy, early GC B cells were gated on single live CD19^+^CD3^−^TLR7a^+^MHC‐II^+^CD95^+^CD38^−^. GC B cells were gated on single live CD19^+^CD3^−^CD95^+^CD38^−^S2^+^ or RBD^+^S1^+^ GC B cells were gated on single live CD19^+^CD3^−^CD95^+^CD38^−^S2^+^ or RBD^+^S1^+^cells. Follicular T cells (Tfh) were gated on single live CD3^+^TCRβ^+^CD4^+^BCL6^+^PD‐1^hi^ cells. Regulatory follicular T cells (Tfr) were gated on single live CD3^+^CD4^+^BCL6^+^PD‐1^hi^FOXP3^+^cells.

For GC‐derived memory B cell analysis, mice were immunized as above (equivalent gardiquimod dose, 20 µg) and dLNs were harvested at week 14. Single‐cell suspensions were prepared and stained on ice for 30 min with RBD and S2 tetramer. After washing and centrifugation, the other surface markers were stained on ice for 30 min, including Fc‐blocker, LIVE/DEAD Fixable Aqua Dead Cell Stain, CD19, CD3, CD80, CD273 (PDL2), CD95, CD38. The gating strategy for GC‐derived memory B cell is shown in Figure 7C.

### Antibody Titer Measurement

Mice were immunized with alum‐adsorbed HexaPro formulated with either TLR7‐Alum or TLR7‐NP (equivalent dose gardiquimod, 20 µg) at tail base at week 0 and 3. Mice were immunized with SARS‐CoV‐2 mRNA vaccine (Pfizer/BioNTech, BNT162b2) at 0.2 µg in 30 µL PBS in the hindlimb at week 0 and 3. Blood samples were collected via facial vein bleeding at weeks 1, 2, 3, and 5 following either the primary or booster immunization. Antibody responses were evaluated by Enzyme Linked Immunosorbent Assay (ELISA) to quantify serum titers against RBD, S2, SARS‐CoV‐2 spike, SARS‐CoV, MERS‐CoV, HCoV‐HKU1, and HCoV‐NL63 antigens. High‐protein‐binding 96‐well plates (Costar) were coated with antigen solutions overnight at 4 °C. RBD, S2, and SARS‐CoV‐2 spike proteins were coated at 5 µg mL^−1^, while the other antigens were coated at 10 µg mL^−1^ in PBS. Plates were washed three times with PBS‐T (PBS containing 0.05% Tween‐20) and then blocked with blocking buffer for 30 min at room temperature. Mouse sera were diluted in blocking buffer and incubated in the wells for 2 h at 37 °C. After three washes with PBS‐T, HRP‐conjugated goat anti‐mouse IgG secondary antibody (Cat. No. 1036‐05, SouthernBiotech, Birmingham, AL, USA) was added at a dilution of 1:5000 and incubated for 1 h. Plates were developed using TMB substrate (Cat. No. N301, Thermo Fisher Scientific, Waltham, MA, USA), stopped with 2 N sulfuric acid, and absorbance was measured at 450 nm using a microplate reader (BioTek Synergy H1, Agilent). Serum from unvaccinated mice was used as the negative control in all ELISA assays.

### Affinity Analysis Via Biolayer Interferometry (BLI)

The apparent K_D_ of serum antibodies collected two weeks after immunization with alum‐absorbed HexaPro combined with either TLR7‐Alum or TLR7‐NP were determined using BLI. Biotinylated S2 protein was immobilized on streptavidin biosensors by loading at 25 nM for 60 s in HBS‐P buffer supplemented with 1% BSA. Serum samples from each group were serially diluted (1:25, 1:50, 1:100, 1:200, and 1:400) in the same buffer and applied to the sensors to monitor association for 600 s, followed by a 600 s dissociation phase in HBS‐P buffer. Measurements were performed using an Octet RED96 system (ForteBio), and data were analyzed using a 1:1 global fit model.

### rVSV‐SARS‐CoV‐2 Neutralization Assay

Vero cells were seeded in DMEM supplemented with 5% fetal bovine serum, 1% penicillin/streptomycin, and 1% GlutaMAX on 96‐well cell culture plates (Corning 3585). Prior to infection, mouse serum was diluted 1:100 followed by threefold serial dilutions in the cell culture media described above. Diluted sera were incubated with a pre‐titrated amount of rVSV‐SARS‐CoV‐2 (Wuhan) for 1 h at room temperature. The media was then removed and replaced with 50 µL virus/sera mixture in duplicate. The plates were incubated with the virus/sera mixture at 37°C and 5% CO_2_ for 8 h. Paraformaldehyde (16%; Cat. No. 043368, Thermo Fisher Scientific, Waltham, MA, USA) was added to a final concentration of 4% to fix the cells, followed by counterstaining of nuclei with Hoechst 33342 (Cat. No. H3570, Invitrogen, Waltham, MA, USA) diluted in PBS (1:5000). Infected cells were quantified by automatic enumeration of GFP‐positive cells from captured images using a Cytation 5 reader (BioTek) with Gen5 software (version 3.12.08). Infection levels were calculated as a percentage of GFP+ cells over the total number of cells and normalized to wells containing no mouse sera. Data were analyzed with GraphPad Prism.

### ELISPOT Analysis

Bone marrow ELISPOT assays were conducted to quantify antigen‐specific antibody‐secreting cells (ASCs) at week 14 following immunization. A detailed protocol was previously published.^[^ ^22^ ^]^ Briefly, Multi‐Screen IP filter plates (0.45 µm, Millipore Sigma) were coated overnight at 4 °C with 50 µL per well of antigen solution in coating buffer. SARS‐CoV‐2 and SARS‐CoV spike proteins were used at 5 µg mL^−1^, while MERS‐CoV and HCoV‐NL63 spike proteins were coated at 10 µg mL^−1^. After coating, plates were washed three times with PBS and blocked with complete DMEM for at least 2 h at 37 °C. Single‐cell suspensions were prepared from bone marrow harvested from the femurs and tibias of immunized mice. Cells were seeded into antigen‐coated plates at a density of 1 × 10^6^ cells per well and incubated at 37 °C for 15 h. After incubation, plates were washed, and an HRP‐conjugated anti‐mouse IgG detection antibody (Cat. No. 1036‐05, SouthernBiotech, Birmingham, AL, USA) was added at a dilution of 1:2500. Antigen‐specific ASCs were visualized using a substrate detection kit (Cat. No. 551951, BD Biosciences, Franklin Lakes, NJ, USA) following the manufacturer's instructions.

### Statistical Analysis

Statistics were analyzed using GraphPad Prism10 software. For the physicochemical characterization study (Figure 1), results are presented as the mean ± standard deviation (SD) from three samples per group. For the B cell activation study (Figure 2), unpaired *t*‐tests with Mann‐Whitney tests were performed, and results are presented as the mean ± SD from four mice per group. For the germinal center (GC) response study (Figure 3), unpaired *t*‐tests with Mann‐Whitney tests were also performed, and results are presented as the mean ± SD from four mice per group. For antibody analyses, unpaired *t*‐tests with Mann‐Whitney or Welch's tests were applied, and data are shown as median values. Except for the samples in Figure 6 (*n* = 9 and 7), all other antibody analyses (Figures 4 and 5) were conducted on vaccinated groups with sample sizes of *n* = 6 and 7. For the durable humoral immunity study (Figure 7), unpaired *t*‐tests with Mann‐Whitney tests were performed, and results are presented as the mean ± SD. The sample sizes for the ELISPOT assay were 7 and 8, respectively, while those for the flow cytometry analysis were four mice per group. Detailed statistical information for each experiment‐including the type of statistical test performed, number of biological replicates, mean values, SDs, and *p*‐values‐is provided in the corresponding figure legends. Statistical significance was defined as ^*^ *p* ≤ 0.05, ^**^ *p* ≤ 0.01, ^***^ *p* ≤ 0.001, ^****^ *p* ≤ 0.0001, ns = not significant.

### Replication of Experiments

All experiments were successfully repeated at least two times.

## Conflict of Interest

Q.Y. and W.L. are inventors on a patent application that describes the use of this nanoparticle for vaccine adjuvant. The remaining authors declare no conflict of interest.

## Author Contributions

S.H., K.M.C., L.C., X.K., C.L., M.D., W.J., A.M., and Z.K. performed experiments. S.H., L.C., M.D., Z.K., K.C., W.L., and Q.Y. designed the experiments, performed data analysis, and contributed to the writing of manuscript. R.T. provided unique research agents. Q.Y. conceptualized, supervised the study, and wrote the initial manuscript. All authors reviewed and approved the manuscript.

## Supporting information



Supporting information

## Data Availability

The data that support the findings of this study are available in the supplementary material of this article.
