# Supplementary material for: Nanoparticle Adjuvant Design Enhances Germinal Center Responses Targeting Conserved Subdominant Epitopes for Pan‐Coronavirus Vaccine Development
Source: Adv Sci (Weinh). 2025 Nov 25;13(7):e12100. doi: 10.1002/advs.202512100 (PMC12866699; doi:10.1002/advs.202512100)
Supplement: Supplementary file 1 — Supporting information [file ADVS-13-e12100-s001.pdf]

Supporting information for

Nanoparticle Adjuvant Design Enhances Germinal Center Responses Targeting Conserved Subdominant Epitopes for Pan-Coronavirus Vaccine Development

Sijin Huang[1], Kanella M. Cohen[1], Liqiang Chen[1], Xiaowo Kang[1], Chang Liu[2], Megan E. Demouth[3], Wenxia Jiang[4], Alexander R. Maldeney[4], Rong Tong[5], Zunlong Ke[2,6], Kartik Chandran[3], Wei Luo[4]*, Qian Yin[1,7]*

[1] Department of Biomedical Engineering, The University of Texas at Austin, Austin, TX, USA

[2] Department of Molecular Biosciences, The University of Texas at Austin, Austin, TX, USA

[3] Department of Microbiology and Immunology, Albert Einstein College of Medicine, New York, NY, USA

[4] Department of Microbiology and Immunology, Indiana University School of Medicine, Indianapolis, IN, USA

[5] Department of Chemical Engineering, Virginia Polytechnic Institute and State University, Blacksburg, VA, USA

[6] LaMontagne Center for Infectious Diseases, The University of Texas at Austin, Austin, TX, USA

[7] Texas Materials Institute, University of Texas at Austin, Austin, TX, USA

*Corresponding author

Lead contact e-mail address: qian.yin@austin.utexas.edu

Reagents

L-Lactide (LLA) was purchased from TCI America (Cat. No. L011525G, Portland, OR, USA). It was recrystallized three times in anhydrous toluene and stored at -30 ^{o}C in glovebox. mPEG-PLGA (LG 50:50 (w:w), Mw 5000:10000Da) was purchased from Akina, Inc. (Cat. No. AK010, West Lafayette, IN, USA). TLR7 agonist (gardiquimod) was purchased from InvivoGen (Cat. No. tlrl-gdq-10 2x5 mg, San Diego, CA, USA). Alexa Fluor^{TM} 647 NHS Ester (Succinimidyl Ester) was purchased from Thermo Fisher Scientific (Cat. No. A20006, Waltham, MA, USA). (BDI)Zn catalysts and precursor were synthesized according to the previous report using similar procedure[1,2]. All other chemical reagents were purchased from Sigma-Aldrich and used as received except where otherwise noted. The commercially available antibodies used in this study were summarized in the following table.

Marker	Company	Cat#	Clone
CD19	BioLegend	152406	1D3
	invitrogen	376-0193-82	eBio1D3
CD3	BioLegend	100216	17A2
CD11c	BioLegend	117343	N418
MHC-II	BioLegend	107622	M5/114.15.2
CD11b	BioLegend	101225	M1/70
PDCA1	BioLegend	127021	927
Ly6C	BioLegend	128041	HK1.4
CD86	BioLegend	105110	P03
CD80	BioLegend	104743	16-10A1
CD95	BD Biosciences	557653	Jo2
CD38	BD Biosciences	740245	90
CD4	BD Biosciences	563790	GK1.5
CD279 (PD-1)	BioLegend	135231	29F.1A12
Foxp3	Invitrogen	17-5773-82	FJK-16s
BCL6	BD Biosciences	561522	K112-91
CD185(CXCR5)	BioLegend	145529	L138D7
CD273 (PDL2)	BioLegend	107215	TY25
TCRβ	BioLegend	109228	H57-597

Animals

8-12 weeks old female C57BL/6 mice were purchased from the Jackson Laboratory. All the animals were housed in the University of Texas at Austin Animal Facility under federal, state and NIH guidelines. The study protocol was reviewed and approved by the University Administrative Panel on Laboratory Animal Care.

Preparation of soluble TLR7 agonist adsorbed with Alum (TLR7-Alum)

The TLR7-Alum was prepared by mixing 25 μL of PBS solution containing 20 μg of the small molecule gardiquimod with Alhydrogel® (V/V, 1/1; Cat. No. vac-alu-50, InvivoGen, San Diego, CA, USA). The mixture was then incubated at room temperature for one hour before use.

Preparation of SARS-CoV-2 HexaPro spike protein formulated with Alum (HexaPro-Alum)

The antigen formulation was prepared by mixing 25 μL of the SARS-CoV-2 HexaPro[3] spike protein solution (10 μg) with Alhydrogel® (V/V, 1/1). The mixture was then incubated at room temperature for 30 minutes prior to use (Fig. S1c).

Preparation of various immunization regimens

For different immunization regimens, the HexaPro spike protein was used either in soluble form or as an alum-adsorbed formulation (HexaPro-Alum) prepared as described above. The TLR7 agonist (Gardiquimod) was used in three formats: soluble (free TLR7), alum-adsorbed (TLR7-Alum), or nanoparticle-formulated (TLR7-NP). Each antigen formulation (HexaPro or HexaPro-Alum) was mixed at a 1:1 volume ratio with the corresponding adjuvant formulation (free TLR7, TLR7-Alum, or TLR7-NP) immediately prior to injection.

Immunization study

For all immunization studies, mice were administered SARS-CoV-2 HexaPro spike protein (10 μg) combined with 20 μg of the TLR7 agonist in the indicated formulation. Each vaccine mixture was prepared in a total volume of 100 μL phosphate-buffered saline (PBS) and administered at the tail base of mice.

Reference

[1] Yin, Q. et al. Pamidronate functionalized nanoconjugates for targeted therapy of focal skeletal malignant osteolysis. Proc Natl Acad Sci U S A 113, E4601-4609 (2016).

[2] Tong, R. & Cheng, J. Ring-opening polymerization-mediated controlled formulation of polylactide-drug nanoparticles. J Am Chem Soc 131, 4744-4754 (2009).

[3] Hsieh et al. Structure-based design of prefusion-stabilized SARS-CoV-2 spikes. Science 369, 1501–1505 (2020).

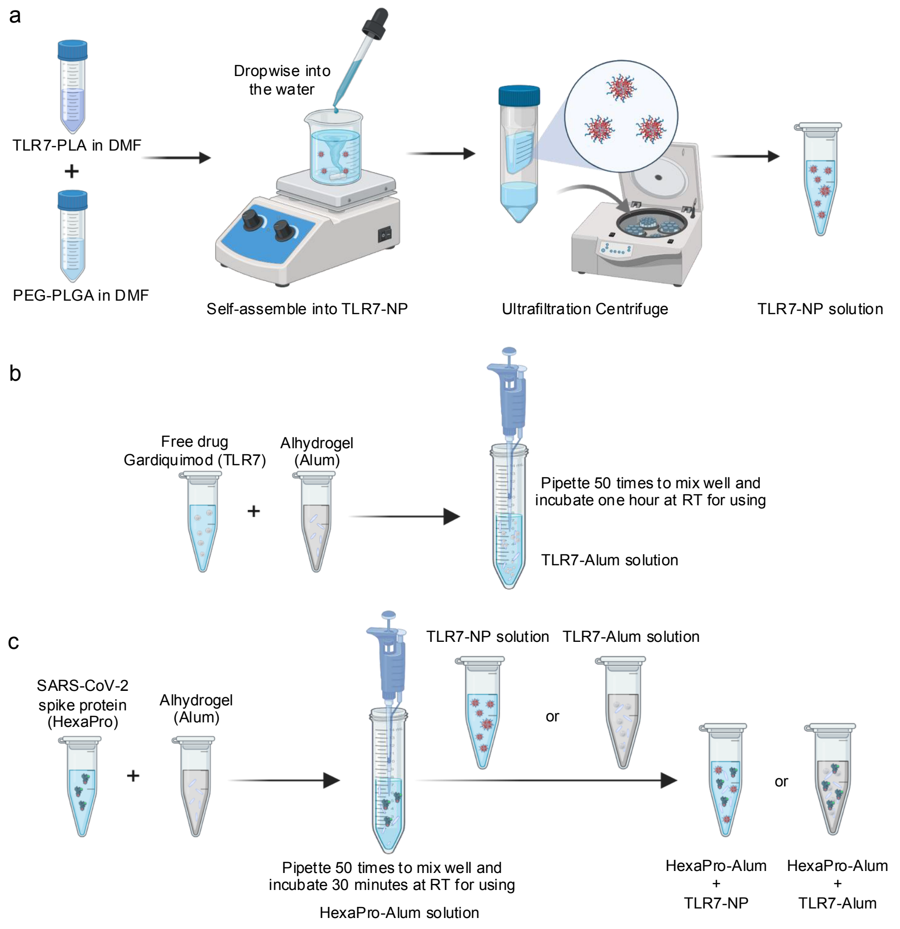

Figure S1. Schematic illustration of the preparation of TLR7-NP (a), TLR7-Alum (b), and HexaPro-Alum+TLR7-NP or HexaPro-Alum+TLR7-Alum (c).

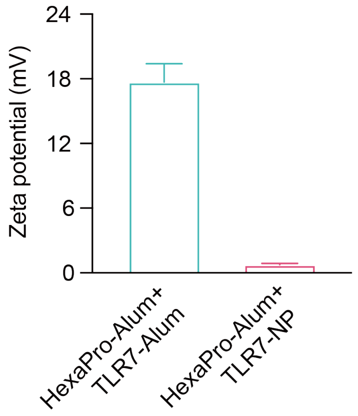

Figure S2. Zeta potential of the vaccine, HexaPro-Alum mixed with adjuvants. Data are represented as mean ± SD (n=3).

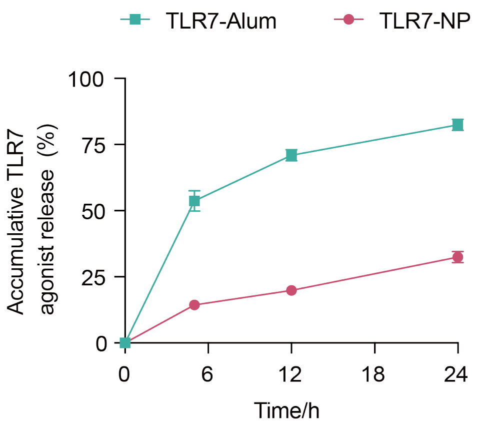

Figure S3. Release kinetics of TLR7 agonist (gardiquimod) from TLR7-Alum and TLR7-NP within 24 hours. Data are represented as mean ± SD (n=3).

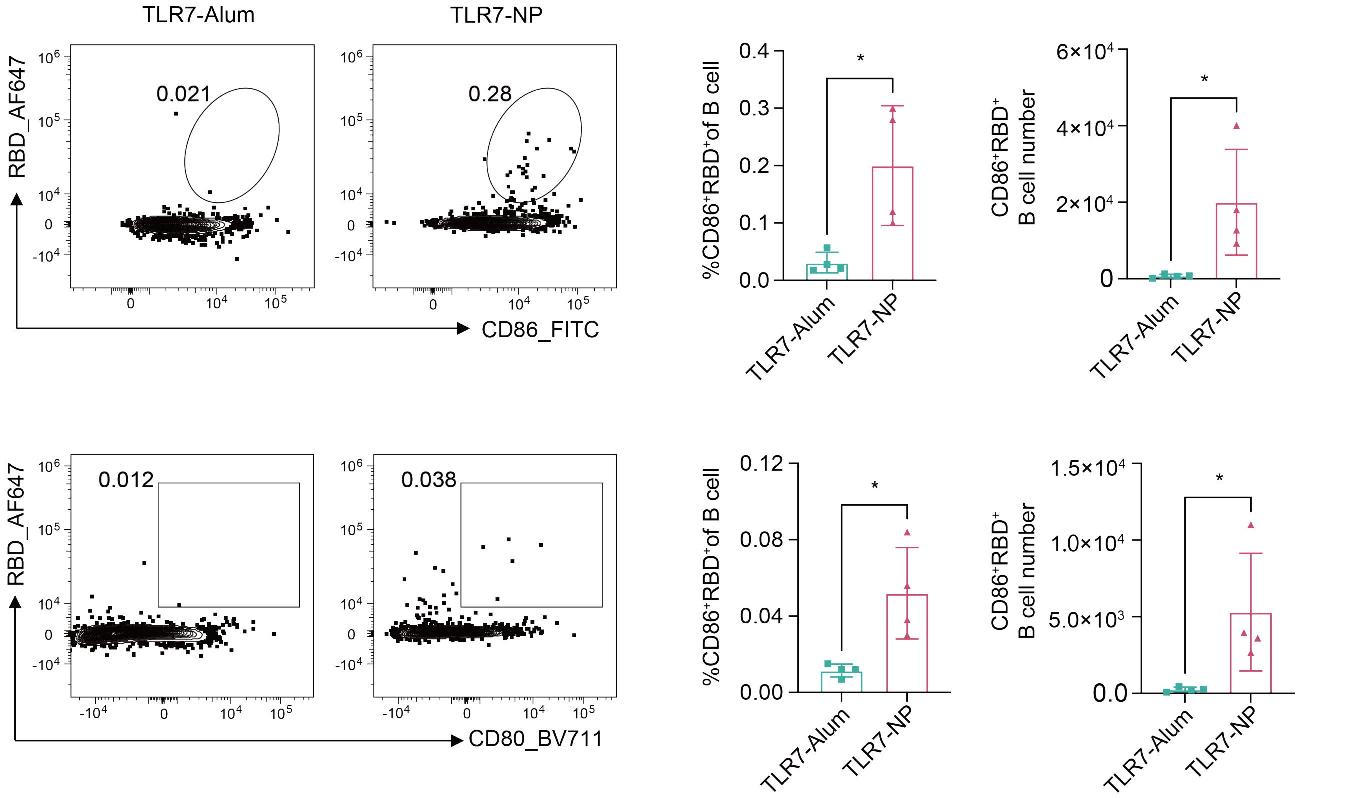

Figure S4. Activation of $RBD^{+}S1^{+}B$ cells analyzed by flow cytometry at day 7. Data are represented as mean ± SD and analyzed by Mann-Whitney test (n=4 mice per group). *p < 0.05, **p < 0.01, ***p < 0.001, ns= not significant.

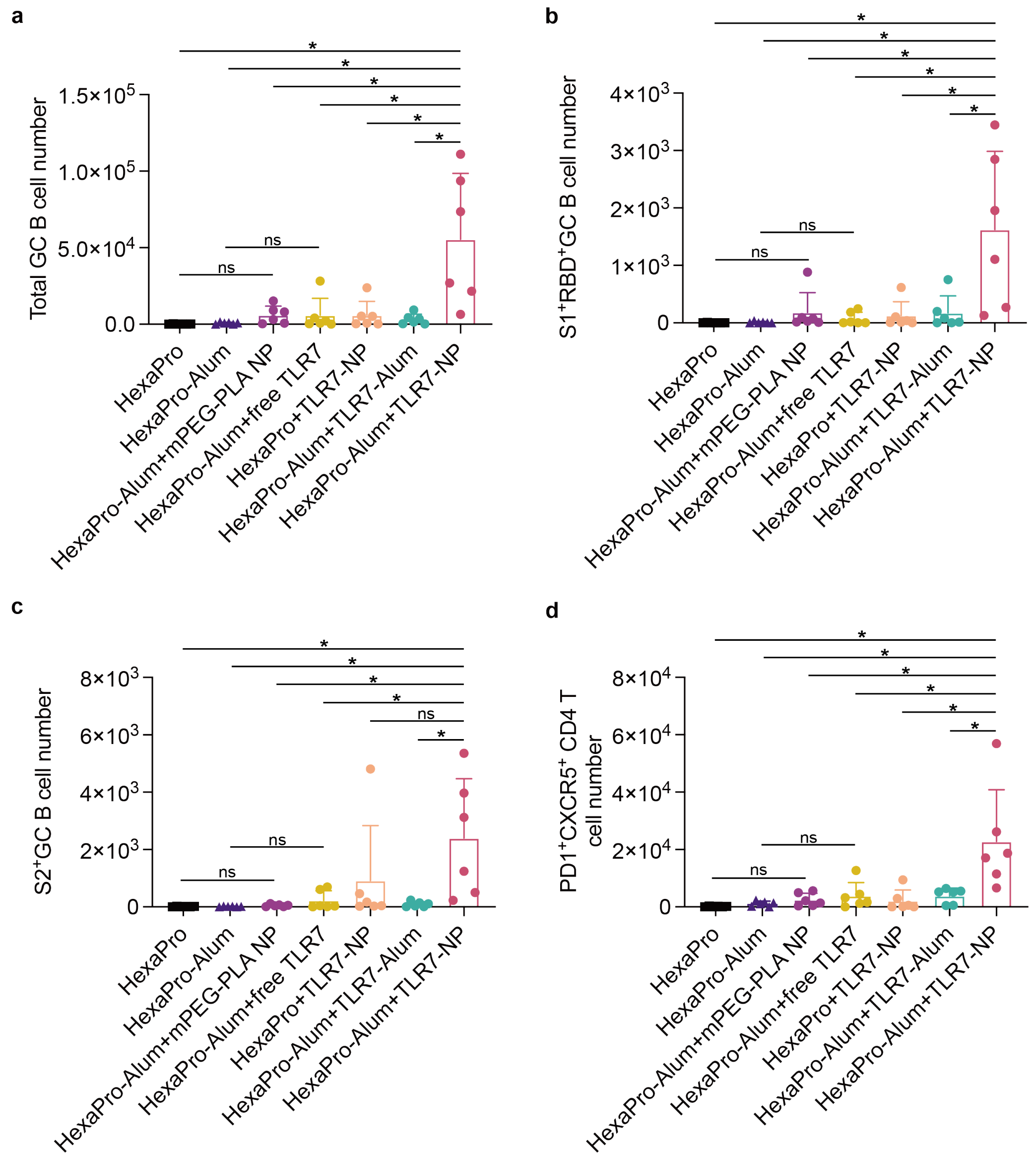

Figure S5. Cell number of GC B cells (a), S1^{+}RBD^{+} GC B cells (b), and S2^{+} GC B cells (c), and PD1^{+}CXCR5^{+} CD4 T cells (d) analyzed by flow cytometry at day 7 for different immunization regimens. Data are represented as mean ± SD and analyzed by Welch's t test (n=6 mice per group, pooled from two independent experiments). *p < 0.05, **p < 0.01, ***p < 0.001, ns = not significant.

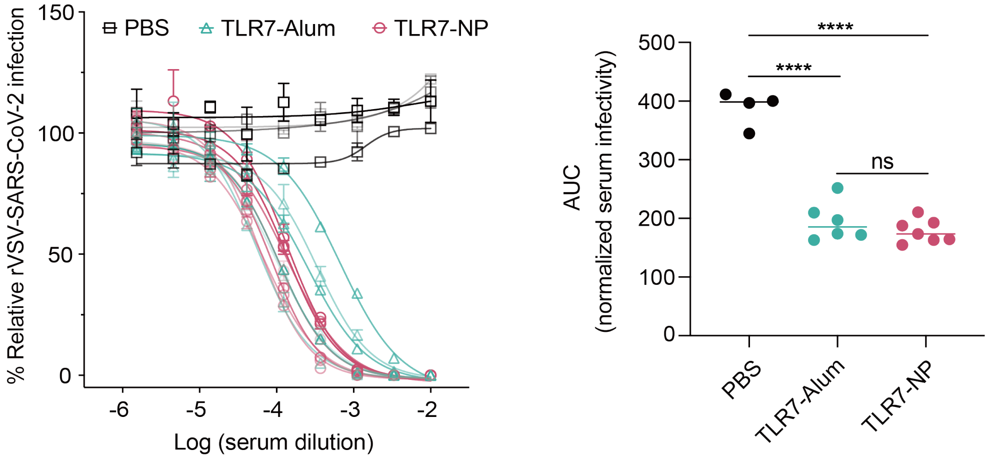

Figure S6. Antibody neutralization assay for 5 weeks of serum. Data are shown as medians with each dot representing one mouse (n = 4, 6, 7 mice for PBS, TLR7-Alum, TLR7-NP group). Data are analyzed by Welch's t test. *$p < 0.05$, **$p < 0.01$, ***$p < 0.001$, ****$p < 0.0001$, ns= not significant.

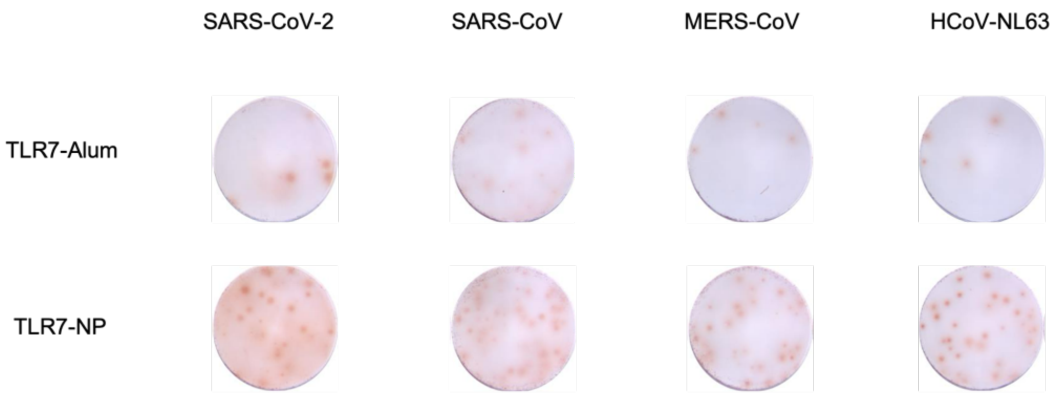

Figure S7. ELISPOT imaging for antibody-secreting cells (ASCs) specific to SARS-CoV-2, SARS-CoV, MERS-CoV, and HCoV-NL63 in the bone marrow at week 14.

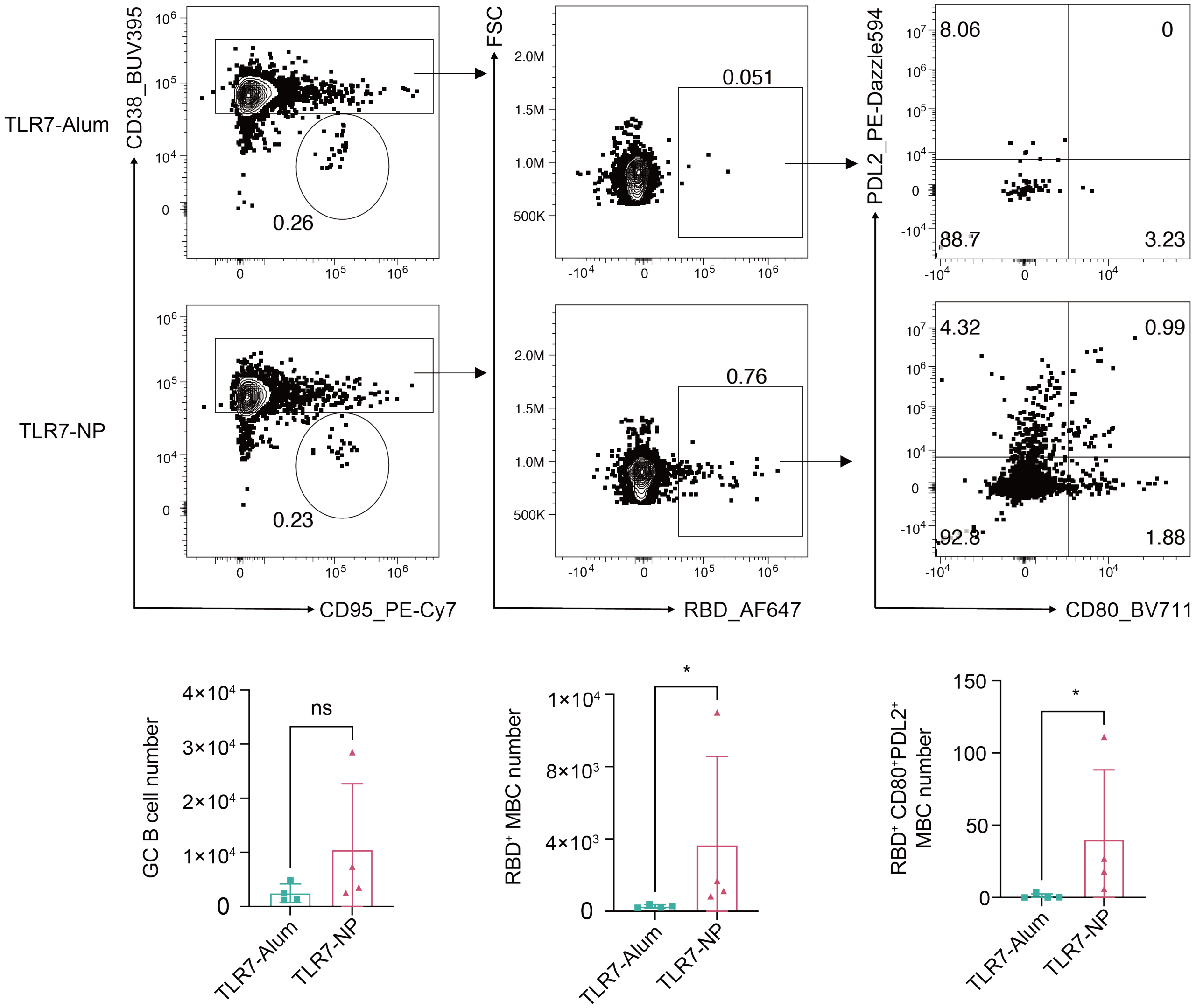

Figure S8. Representative flow plots and quantification of RBD-specific memory B cell (RBD^{+}MBC) and its $CD80^{+}PDL2^{+}$ subsets ($RBD^{+}CD80^{+}PDL2^{+}$MBCs) in dLNs. Data are analyzed by Mann-Whitney test. *$p < 0.05$, **$p < 0.01$, ***$p < 0.001$, ns = not significant. (n=4 mice per group)
